# Ageing-associated DNA methylation dynamics are a molecular readout of lifespan variation among mammalian species

**DOI:** 10.1186/s13059-018-1397-1

**Published:** 2018-02-16

**Authors:** Robert Lowe, Carl Barton, Christopher A. Jenkins, Christina Ernst, Oliver Forman, Denise S. Fernandez-Twinn, Christoph Bock, Stephen J. Rossiter, Chris G. Faulkes, Susan E. Ozanne, Lutz Walter, Duncan T. Odom, Cathryn Mellersh, Vardhman K. Rakyan

**Affiliations:** 1 The Blizard Institute, Barts and The London School of Medicine and Dentistry, Queen Mary University of London, London, E1 2AT UK; 2 Kennel Club Genetics Centre, Animal Health Trust, Newmarket, Suffolk, CB8 7UU UK; 3 Cancer Research UK Cambridge Institute, University of Cambridge, Cambridge, CB2 0RE UK; 4 University of Cambridge Metabolic Research Laboratories and MRC Metabolic Diseases Unit, Wellcome Trust-MRC Institute of Metabolic Science, Addenbrooke’s Hospital, Cambridge, CB2 0QQ UK; 5 CeMM Research Center for Molecular Medicine of the Austrian Academy of Sciences, Vienna, Austria; 6 Department of Laboratory Medicine, Medical University of Vienna, Vienna, Austria; 7 Max Planck Institute for Informatics, Saarland Informatics Campus, Saarbrücken, Germany; 8 Ludwig Boltzmann Institute for Rare and Undiagnosed Diseases, Vienna, Austria; 9 School of Biological & Chemical Sciences, Queen Mary University of London, Mile End Road, London, E1 4NS UK; 10 Primate Genetics Laboratory, Leibniz Institute for Primate Research, German Primate Center, Göttingen, Germany; 11 Centre for Genomic Health, Queen Mary University of London, EC1M 6BQ, London, UK

**Keywords:** Ageing, Methylation, Epigenetics

## Abstract

**Background:**

Mammalian species exhibit a wide range of lifespans. To date, a robust and dynamic molecular readout of these lifespan differences has not yet been identified. Recent studies have established the existence of ageing-associated differentially methylated positions (aDMPs) in human and mouse. These are CpG sites at which DNA methylation dynamics show significant correlations with age. We hypothesise that aDMPs are pan-mammalian and are a dynamic molecular readout of lifespan variation among different mammalian species.

**Results:**

A large-scale integrated analysis of aDMPs in six different mammals reveals a strong negative relationship between rate of change of methylation levels at aDMPs and lifespan. This relationship also holds when comparing two different dog breeds with known differences in lifespans. In an ageing cohort of aneuploid mice carrying a complete copy of human chromosome 21, aDMPs accumulate far more rapidly than is seen in human tissues, revealing that DNA methylation at aDMP sites is largely shaped by the nuclear trans-environment and represents a robust molecular readout of the ageing cellular milieu.

**Conclusions:**

Overall, we define the first dynamic molecular readout of lifespan differences among mammalian species and propose that aDMPs will be an invaluable molecular tool for future evolutionary and mechanistic studies aimed at understanding the biological factors that determine lifespan in mammals.

**Electronic supplementary material:**

The online version of this article (10.1186/s13059-018-1397-1) contains supplementary material, which is available to authorized users.

## Background

The large variation in lifespan among different mammalian species is a fascinating yet poorly understood phenomenon. For example, mice, on average, live for only two years, whereas other species such as humans and whales can live for > 100 years. Thus far, a variety of different factors have been proposed to correlate with mammalian species lifespan such as body mass, metabolic rate and age of menarche (reviewed in [1]). However, in each case there are exceptions leading to confusion. For example, although body mass is positively correlated with lifespan across mammalian species, this relationship is not true within species such as dogs [2]. Furthermore, these factors are not dynamic molecular correlates of lifespan.

Recently, several studies have reported genome-scale profiles of ageing-associated differentially methylated positions (aDMPs) in the human genome – CpG sites at which DNA methylation dynamics shows a significant correlation with age (a few of the papers in this area are listed in the references [3–8]). Currently, aDMPs represent the most accurate known molecular markers of age in humans. aDMPs are not limited to humans, with other recent studies showing similar effects in mice [9–14] and whales [15]. In the former, it was also observed that lifespan-altering interventions can change the rate of ageing-associated DNA methylation dynamics at aDMPs [10–14]. Inspired by these recent results, we hypothesised that not only are aDMPs pan-mammalian, but they could also represent the first known dynamic molecular readout of lifespan variation among different mammalian species.

## Results

### The rate of change of ageing-associated DNA methylation is faster in the mouse relative to human

**Fig. 1:**
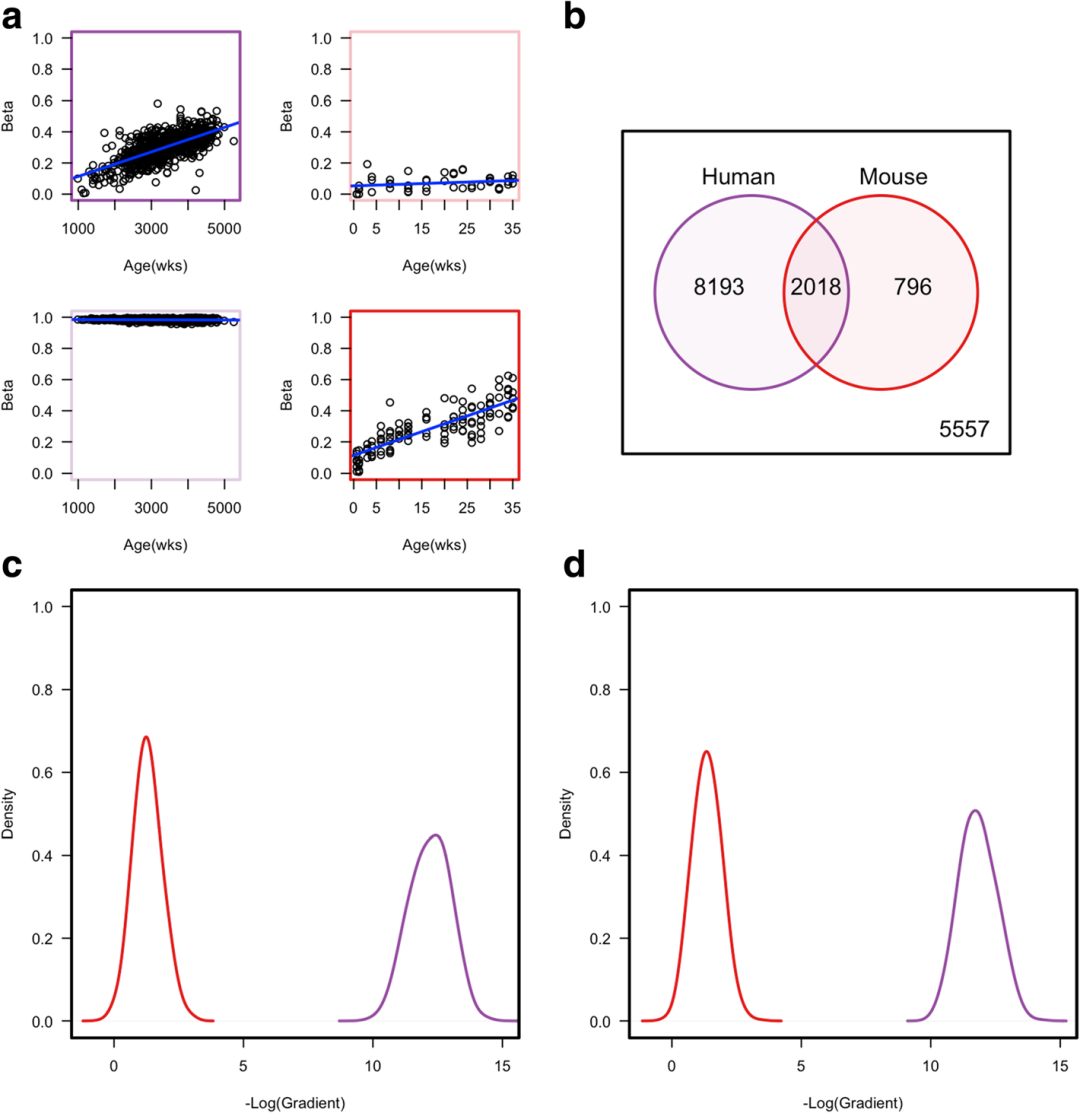
**a** Example of called aDMPs. *Top:* A significant aDMP in human samples (*top left*) but not in mouse samples (*top right*). *Bottom:* A significant aDMP in mouse samples (*bottom right*) but not in human samples (*bottom left*). Thick coloured *boxes* represent a genome-wide significance aDMP in either human (*purple*) or mouse (*red*). **b** A *Venn diagram* representing the overlap between called aDMPs in mouse and human. **c** A *density plot* of the negative log transformed gradients for those aDMPs in non-sequence conserved regions in either mouse (*red*) or human (*purple*). **d** A *density plot* of the negative log transformed gradients for those aDMPs in regions showing sequence conservation between mouse (*red*) and human (*purple*)

We first re-analysed published ageing-associated genome-scale methylation datasets for human and mouse. This involved calling ageing-associated differentially methylated positions (aDMPs) from array-based Illumina 450 K data for 656 human samples from Hannum et al. [3] and Reduced Representation Bisulphite Sequencing (RRBS) data for 153 mice from Petkovich et al. [11] (see ‘Methods’). At q-value < 0.01, we found 172,365 CpGs in human and 43,909 CpGs in the mouse that were called aDMPs. Analysis of conserved CpG sites only revealed that approximately 70% were called as aDMPs in both species, suggesting that short-range *cis* sequence is unlikely to be the only driving factor in determining whether any given CpG site behaves as an aDMP (Fig. 1b). As mice have a considerably shorter lifespan than humans, the ability to detect aDMPs in mice suggests that the rate of DNA methylation change must be considerably faster. For example, in Fig. 1a, the mouse aDMP shows a methylation change of ~ 50% that occurs within just 35 weeks. To confirm this was a general feature of detected aDMPs, we calculated the rate of change of methylation per week for both human and mouse aDMPs and found that irrespective of whether we use conserved or non-conserved aDMP sites, mouse aDMPs showed a significantly (*P* value < 2.2 × 10^–16^) faster rate of change than human aDMPs (Fig. 1c, d), consistent with Stubbs et al. [10]. One potential caveat when comparing species with very different lifespans such as mouse and human is that if there are mouse aDMPs which show similar slow rates of methylation dynamics to those of human aDMPs, they would be difficult to detect as their dynamics would be much too slow to be detected within the lifetime of a mouse. Nevertheless, we can confidently state that at least a significant proportion of aDMPs show considerably different dynamics between a short-lived (mouse) and long-lived (human) mammalian species.

### aDMP dynamics are related to mammalian species lifespan

**Fig. 2:**
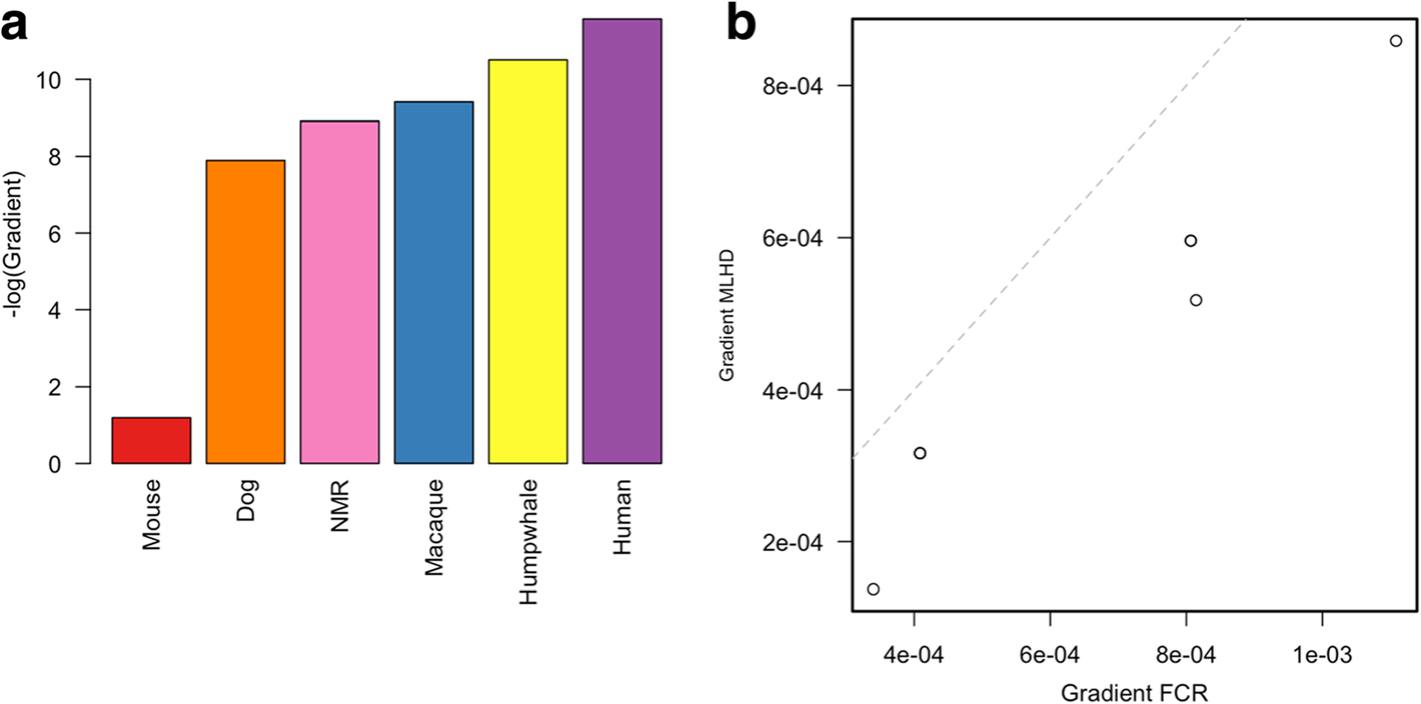
**a** A *bar plot* of the mean negative log gradients for six different species. This shows that the rate of methylation change at aDMPs is proportional to the longevity of the species. **b** A *plot* showing the gradient of significant aDMPs for the two dog breeds profiled (FCR and MLHD). In each of the six aDMPs (two points have similar gradients and overlap each other), the gradient of the FCR is larger than that of the MLHD. *Dashed line* is the line which represents equal gradient in both breeds, e.g. y = x

To extend the above findings, we investigated aDMP methylation dynamics in six different mammalian species spanning a range of documented maximum lifespans (T_*max*_), which is a commonly used estimate for the rate of ageing: mouse (*Mus musculus,* T_*max*_ = 4 years), dog (*Canis familiaris*, T_*max*_ = 24 years), naked mole rat (NMR) (*Heterocephalus glaber,* T_*max*_ = 31), rhesus macaque (*Macaca mulatta,* T_*max*_ = 40), humpback whale (*Megaptera novaeangliae,* T_*max*_ = 95) and human (*Homo sapien,* T_*max*_ = 122) (all T_*max*_ values are from ‘*AnAge*’, http://genomics.senescence.info/species). Due to the high cost of generating sequencing-based deep coverage genome-scale methylation data across many samples, and given that cost-effective commercial DNA methylation arrays are only available for human, we analysed 48 different targeted bisulphite polymerase chain reaction (Bis-PCR) sequencing amplicons for dog and NMR that were chosen based on sequence conservation with human aDMP sites (we show above that human vs mouse differences in methylation ageing rates hold for both conserved and non-conserved aDMP sites). For macaque, due to its close evolutionary distance to human, we generated aDMP profiles using the Illumina 450 K array (previous studies have successfully used this cost-effective microarray platform for assaying genome-scale methylation differences in a variety of primate species [16, 17]). For humpback whale, we used existing targeted Bis-PCR sequencing data from Polanowski et al. [15]. For human and mouse, we used previously published genome-scale datasets [3, 14]. Detailed sample statistics are available in Table 1. For dog, we identified 68 aDMPs that clustered in 15 different targeted aDMP regions (adjusted *P* value < 0.05) and for NMR we identified 30 aDMPs that clustered in 11 different targeted aDMP regions (adjusted *P* value < 0.05). For macaque, we determined 29 distinct aDMP regions (*P* value < 5 × 10^–5^). From each of these CpGs, we determined the rate of dynamic change in methylation levels per week for each species. This yielded a significant negative correlation between rate of change of methylation at aDMP sites and reported T_*max*_ across the different species (*rho* = 1, *P* = 0.0028, Spearman correlation) (Fig. 2a note that in this figure we plot the ‘–log gradient’ of methylation ageing rate). There are four key points to note about these findings: (1) this relationship holds even when comparing mammalian species such as dog, NMR and rhesus macaque, i.e. species with more similar T_*max*_ values to each other, compared with the extreme differences between mouse and human; (2) this relationship is with lifespan per se and not confounded with body mass differences as aDMP methylation dynamics are faster in whales relative to humans even though the former is a much bigger species in terms of mass; (3) our use of different tissues across the different species has negligible influence on the relationship with mammalian lifespan we report here, as analysis of previously published aDMP data from various human tissues reveals that they display similar rates of change with age, and these are significantly greater than between-species differences we report here (Additional file 1: Figure S1); (4) although females show slightly slower ageing-associated methylation dynamics relative to males in human [3], this difference is again smaller than the differences we find among mammalian species.

**Table 1:** Sample information for those used in the paper

Species	n	Tissue	Age range (weeks)	Reference	Platform	aDMRs (n)^a^
Human	656	Blood	988–5252	Hannum et al.	450 K	256
Mouse	153	Whole Blood	0.67–35	Petkovich et al.	RRBS	2814
Dog	48	Buccal	13–726.96	Own	Bis-PCR	15
NMR	24	Liver	39–1144	Own	Bis-PCR	11
Macaque	6	Blood	52–1040	Own	450 K	29
Humpback whale	45	Skin	2.6–1576.953	Polanowski et al.	Qiagen PyroMark assays	3
Tc1	6	Liver	8–52	Own	450 K	-

^a^The number of aDMRs is the number of aDMRs used in Fig. 2a

### The correlation between rate of change at aDMPs and lifespan is observed between two different dog breeds

To examine if the negative correlation between rate of change of methylation at aDMP sites and reported lifespan also holds within a species, we analysed two different dog breeds. Dogs have lived alongside humans for thousands of years and shared similar environmental influences. Artificial selection has led to the generation of > 200 varieties (‘pure breeds’) with strikingly different but well characterised phenotypes and attributes, including lifespan which can be studied outside of artificial laboratory conditions. We examined two different pure breeds with contrasting lifespan – the miniature long-haired dachshund (MLHD) (average life expectancy of 12–15 years) and flat-coated retriever (FCR) (average life expectancy of 8–10 years) ([18] and www.thekennelclub.org.uk/pedigreebreedhealthsurvey)). Only animals that were disease-free at time of sampling were included in our analysis. From the 15 different dog aDMP regions, six regions were identified as aDMPs in both breeds. For all six of these aDMP regions, we found that the shorter-lived FCR showed a significantly faster rate of change of methylation relative to the longer-lived MLHD (*P* value = 0.0068, Wilcoxon rank-sum test) (Fig. 2b). This difference remains unchanged even after removing animals that were aged < 2 years (a conservative estimate of sexual maturity in dogs). Overall, this provides an example of the negative relationship between rate of change of methylation at aDMP sites and lifespan within a mammalian species.

### Rate of change of aDMPs is related to the cellular milieu

**Fig. 3:**
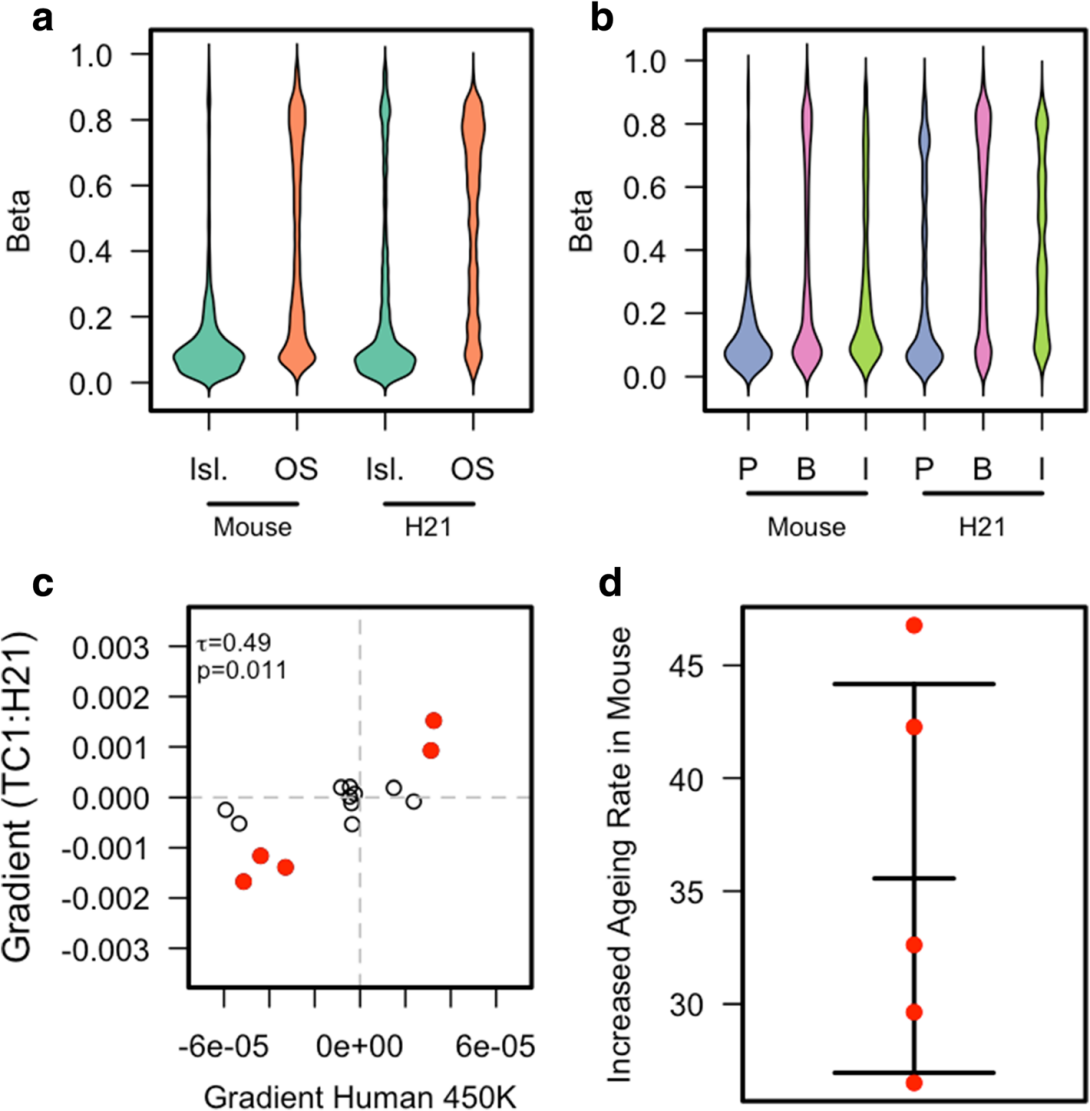
**a** A *bean plot* of the methylation levels for CpG Islands (Isl.) and Open Sea (OS) for both probes matching mouse genome and those uniquely mapping to human chromosome 21. **b** A *bean plot* of the methylation levels for promoters (P) (defined as 2 kbp upstream from the TSS), gene bodies (B) and intergenic regions (I) (defined as those probes not either in a promoter or gene body). This is shown for both probes matching mouse genome and those uniquely mapping to human chromosome 21. **c** A *scatter plot* of the gradients of human chromosome 21 aDMPs in both human samples and the TC1 mouse. There is a significant correlation of 0.49 (*P* value = 0.011). Highlighted in *red* are the probes which show the largest gradient in the TC1 mouse. **d** A *scatter plot* showing the increased rate of methylation change with respect to age in the human chromosome 21 aDMPs in the Tc1 mouse compared to the same aDMP in human samples

Given the lack of conserved aDMPs across species, it is unlikely that short-range cis-sequence plays a major role. Rather, we hypothesised that the ageing cellular environment per se influences aDMP methylation dynamics. Since this cannot be addressed just by comparing different mammalian species, we used a transchromosomic mouse strain – ‘Tc1’ – that harbours a freely segregating and largely intact functional human chromosome 21 (h-chr21) [19]. We profiled the DNA methylation of 8- to 12-week-old and 44- to 52-week-old Tc1 mice using the human Illumina 450 K array. This allowed us to determine the methylation state of 3158 CpGs on h-chr21. We restricted our analysis to h-chr21 probes that have no significant sequence similarity in the mouse, hence minimising any cross-hybridisation artefacts associated with aDMPs. Furthermore, it has been previously shown that 13,715 CpGs on the 450 K array also bind mouse DNA [20]. Since the DNA samples only contained human DNA from chromosome 21, we removed those probes from human chromosome 21 that also robustly bind mouse DNA, allowing us to confidently assay the methylation state of 12,358 CpGs in the mouse genome and thus permitting a valuable control analyses. A number of previous studies have shown that the majority of functional attributes of h-chr21in the Tc1 mouse are similar to those found in human cells [21]. Indeed, we confirmed that both mouse and h-chr21CpGs showed lower methylation levels in CpG Islands and promoters compared to open sea, gene bodies and intergenic regions (Fig. 3a, b). Given the relatively small number of Tc1 mice available for our analyses, we did not have the statistical power to perform *de novo* aDMP calling, but we were able to compare previously reported aDMP profiles with methylation differences between young and old TC1 mice. We therefore initially attempted to investigate the aDMP signature on h-chr21 using existing human liver data (*n* = 117) ([22] and GSE61258) but found only four aDMPs. Therefore, to provide a more robust set of aDMPs we called pan-tissue aDMPs using samples from multiple human tissues (*n* = 350) [23]. This yielded 15 aDMPs on human chr 21. Analysis of these in the Tc1 mouse revealed a significant correlation of directionality in ageing-associated dynamics between aDMPs on human chr21 in human cells and the corresponding sites on h-chr21 in the Tc1 mouse (*P* = 0.011, Fig. 3c). More strikingly, fitting a linear model across all 15 points revealed that the rate of change of methylation with age at these chr21 aDMP sites is approximately 21 times faster in the mouse relative to what is observed in humans (note the differences in scale between the x- and y-axes in Fig. 3c). Calculating the methylation rate of change for just those aDMPs with the largest change in the Tc1 mouse showed a mean increased rate of ~ 35 times that of the same aDMP in humans (Fig. 3d). Therefore, aDMPs on a chromosome from a long-lived species (human) show greatly accelerated methylation dynamics in a short-lived species (mouse), demonstrating that such aDMPs are measuring the rate of cellular ageing.

## Discussion and conclusions

Here we have shown that ageing-associated DNA methylation is a dynamic correlate of lifespan among mammalian species and that these methylation dynamics are measuring cellular ageing as opposed to just chronological age. Crucially, our work shows that, in the context of different mammalian species, the rate of methylation dynamics at aDMPs predict lifespan. That is, aDMPs are more than just a measure of chronological age. Although recent studies have shown that lifespan-altering interventions are associated with changes in dynamics of the epigenetic ageing clock, our work [10–14], in particular the Tc1 experiments, highlights the significant extent to which the dynamics of the clock can be modulated by the trans-nuclear environment. It is also important to note that in recently published comparisons of human with mouse [10–14] and/or macaque epigenetic clocks [24], it was impossible to determine whether the differences in aDMP dynamics among the different species were due to lifespan or body mass differences. On the other hand, in our manuscript we can categorically state that the rate of the epigenetic clock is a measure of lifespan per se and not body mass.

Our work raises two key questions that need to be addressed in future research. First, what is the biological basis of the link between aDMPs and mammalian lifespan? The data from the Tc1 mice show that aDMP dynamics are a consequence of cellular ageing. Ageing comprises a multitude of different processes and one could speculate that the overall aDMP signature reflects information integrated from different sources, as opposed to a single cause. These different upstream processes have stable, cumulative (and measurable) influences on independent subsets of CpG sites, that collectively represent the aDMP signature. Such a model is also consistent with the idea that of the few known factors thought to influence mammalian lifespan, including body mass, none can solely explain aDMPs. It would be interesting to specifically modulate known signalling pathways to establish how they alter dynamics at specific aDMPs. In this regard, it will be worth exploring whether aDMPs are also found in non-mammalian species that harbour DNA methylation (such as many insects), as these organisms typically have much shorter lifespans and hence provide a more tractable system. Second, it will be important to investigate the functional impact of aDMPs. Within this context, relevant questions include whether there are broader changes in epigenetic state, and/or gene expression, and how such changes might influence ageing-associated processes. Recent large-scale integrative analyses suggest only modest correlations between ageing-associated DNA methylation and gene expression dynamics [3, 23]. This is largely consistent with the complex relationship between DNA methylation and gene expression levels, and it may be that ageing-associated DNA methylation dynamics influence the response of a gene as opposed to steady-state levels. Recent advances in epigenetic engineering methodologies may allow the creation of aDMP signatures in cells (e.g. [25]), thus enabling a more direct assessment of their function including effects on gene expression.

Although future research will undoubtedly address the questions discussed above, it is already clear that aDMPs are a readout of mammalian cellular ageing and, to the best of our knowledge, the first dynamic molecular correlate of lifespan differences among mammalian species. Consequently, they have great potential as molecular markers for studying evolutionary and mechanistic aspects of mammalian ageing.

## Methods

### DNA samples

We generated DNA methylation data for dogs, NMRs, macaques and Tc1 mice. All animals did not show any obvious signs of disease at time of sampling. NMRs were maintained at Queen Mary University of London in the Biological Services Unit, in compliance with institutional guidelines. Macaques were maintained at the German Primate Center, Leibniz-Institute for Primate Research, as a self-sustaining colony of rhesus macaques (Macaca mulatta).

### Data processing

For the 656 samples from Hannum et al., preprocessed samples were downloaded from GEO with accession (GSE40279). For 153 mice from Petkovich et al. [11], methylation and coverage values for each CpG was obtained from GEO with accession GSE80672. Those CpGs with < 50× coverage in a minimum of 133 samples were removed from the analysis. For Dog and NMR samples, raw FASTQ files were mapped to the reference canFam3 and hetGla2, respectively, using BISMARK (v0.16.3) [26] and Bowtie2 (v2.2.8) [27]. Reads that mapped outside of the targeted regions were discarded from analyses and methylated and unmethylated counts for each CpG were calculated using the custom C++ program (https://bitbucket.org/lowelabqmul/methylation-extractor). Those CpGs with a coverage < 50× were also discarded from analyses. For Macaque samples, we first mapped 450 K probe sequences to the rheMac8 reference genome using BWA [28]. Probes with no mismatch within the first 5 bp and with < 3 mismatches in total were kept for further analysis (125,102 probes). From these probes, those with a detection *P* value > 0.01 were removed and a quantile normalisation on the Red, Green and Type II probes was performed. For TC1 mouse samples, a list of probes previously shown to map to mouse genome was used as well as those probes mapping to h-chr21. Additional filtering was performed to remove probes mapping to h-chr21 which are either deleted or duplicated. Processing was then performed in the same manner as macaque. For Humpback whale, we downloaded methylation values from DRYAD (http://hdl.handle.net/10255/dryad.58061). Data created for this manuscript is available from GEO with accession number GSE86059. Primers used for targeted bisulphite sequencing are listed in Additional file 2.

### aDMP calling

For all aDMP calling, we used the dmpFinder function available in the R package [29] minfi [30] with default parameters, using age in weeks as phenotype variable and continuous as the type. q-values was calculated using the positive false discovery rate [31]. This provided *P* values and q-values used to filter aDMPs as well as the gradient from the fitted linear model. aDMPs regions were defined by extending each aDMP by ± 100 bp. Then, overlapping regions are combined into a single region spanning the entire length of the extended aDMPs and the maximum gradient and minimum *P* value are assigned to the region. Therefore, each region will be a minimum of 200 bp if only a single aDMP is contained in it.

### Sequence conservation analysis

For comparing between species, we used the UCSC tool liftOver [32] with default parameters except for setting the minimum ratio of bases that must remap to 90% using the parameter ‘-minMatch = 0.9’. To liftOver 450 k probes, we defined a region of 200 bp (± 100 bp) around the target CpG.

## Additional files

Additional file 1: Figure S1. Barplot of the negative log gradient of age in weeks against methylation for multiple different tissues from human samples. (DOCX 113 kb)

Additional file 2: A table of primer sequences used for targeted assay of methylation. (CSV 3 kb)
