# Supplementary material for: Ageing-associated DNA methylation dynamics are a molecular readout of lifespan variation among mammalian species
Source: Genome Biol. 2018 Feb 16;19:22. doi: 10.1186/s13059-018-1397-1 (PMC5815211; doi:10.1186/s13059-018-1397-1)
Supplement: Supplementary file 1 — Barplot of the negative log gradient of age in weeks against methylation for multiple different tissues from human samples. (DOCX 113 kb) [file 13059_2018_1397_MOESM1_ESM.docx]

**Figure S1:** The negative log gradient of multiple different tissues from human samples. There is very little difference between the gradients measured. Liver samples are from TCGA and GSE61258 (n=117), Breast sampled are from TCGA (n=98), Kidney samples are from TCGA (n=204) and Blood samples are from Hannum et al. as used in the paper.
